# Rheology of Conductive High Reactivity Carbonaceous Material (HRCM)-Based Ink Suspensions: Dependence on Concentration and Temperature

**DOI:** 10.3390/nano13010021

**Published:** 2022-12-21

**Authors:** Claudia Dessi, Nicola Melis, Francesco Desogus, Luca Pilia, Roberto Ricciu, Massimiliano Grosso

**Affiliations:** 1 Clermont Auvergne INP, ICCF, Université Clermont Auvergne, F-63000 Clermont-Ferrand, France; 2 Department of Mechanical, Chemical and Materials Engineering, University of Cagliari, 09123 Cagliari, Italy; 3 Department of Environmental Civil Engineering and Architecture, University of Cagliari, 09123 Cagliari, Italy

**Keywords:** graphene, HRCM, inks, suspensions, viscosity, temperature dependence, non-Newtonian flow, sheet resistance

## Abstract

The present case study reports a shear rheological characterization in the temperature domain of inks and pastes loaded with conductive High Reactivity Carbonaceous Material (HRCM) consisting mainly of few-layers graphene sheets. The combined effect of filler concentration and applied shear rate is investigated in terms of the shear viscosity response as a function of testing temperature. The non-Newtonian features of shear flow ramps at constant temperature are reported to depend on both the HRCM load and the testing temperature. Moreover, temperature ramps at a constant shear rate reveal a different viscosity–temperature dependence from what is observed in shear flow ramps while maintaining the same filler concentration. An apparent departure from the well-known Vogel–Fulcher–Tamman relationship as a function of the applied shear rate is also reported.

## 1. Introduction

Two-dimension (2D) particle suspensions have become a research topic of immense interest worldwide, given their large potential applications in many important fields such as advanced heat transfer [1], energy harvesting [2], medical systems, microfluidics, and microelectronics [3]. Due to the large benefits compared to suspensions with one-dimensional (1D) particles and conventional fluids, most studies have mainly focused on the determination, modelling, and simulating material properties, such as thermal and electrical conductivity, of 2D particle suspensions in static conditions. In particular, this has been carried out for nanomaterials filled with carbonaceous fillers (e.g., graphene and graphene oxide), hexagonal boron nitride, and molybdenum disulfide as 2D particles [4,5,6,7]. However, when it comes to practical applications where the composite system is made to flow, dynamic viscosity and the related flow stress become very important properties to evaluate as well. They, indeed, may set the optimal conditions at which crucial physical properties of the material are ensured to be homogeneous and have high performance while going under flow (e.g., convective heat transfer in flow and any flow processing performance) or at rest (e.g., the electrical conductivity of printed microelectronics) [8,9,10,11]. 

In the particular case of 2D particle suspensions and molten composites, several works report either Newtonian or non-Newtonian flow behavior, thixotropicity, macroscale superlubricity, capability of forming particle network under shear flow, the presence of negative-to-positive normal stress first difference, and rheological responses similar to rod-like liquid crystal polymers as the main under-flow features [12,13,14,15,16,17,18,19,20,21,22,23,24,25,26]. Except for the work by Arapov and coworkers [22], the aforementioned studies have been carried out at a single temperature (room temperature in most cases) or within a small range of temperatures. However, temperature and its effect on viscosity are of great importance to predict the flow behavior of suspensions at different particle concentrations. In the review by Murshed and Estelle, the state of the art of temperature influence on viscosity for particle suspensions shows very contrasting results [27]. At a fixed particle concentration, the relative viscosity decrease or invariance with temperature increment has often been observed for 1D particle suspensions [28,29,30] and 2D particle suspensions as well [19,20,25,26,31,32]. On the other hand, studies by Kole and Dey [33] and Sundar et al. [34] reported an inverse temperature effect on the viscosity of 1D particle suspensions (i.e., viscosity increase with temperature increase), particularly at 50 °C or slightly higher temperatures. The results are scattered, but the observed increase in viscosity was considerable. To our knowledge, the latter behavior has not yet been reported for 2D particle suspensions.

An ever-emerging technical application for 2D particle suspensions is represented by the printing sector [35]. In this field, the filled suspension systems may show even more complex dynamics in flow which in turn may affect crucial technical properties. For instance, suspensions filled with conductive particles, such as conductive inks, of regardless liquid- or paste-like texture, should result in homogeneous strips having enough conductive particles to ensure high conductivity and mechanical stability after both the printing and the solvent-drying processes. Successful conductive ink formulations for printing have been shown to have values of sheet resistance on the order of magnitude of 10 to 100 Ω sq^−1^ with final applications spanning from thin-film transistors to transparent electrodes, respectively [36,37,38,39]. To make the production of these materials even more sustainable, employing a low-environmental-impact solvent and a mass-production-scalable filler for the ink formulation, is highly recommended.

Here we present a study of shear rheological characterization in the temperature domain of conductive ink suspensions intended for printed electronics and IoT applications. The suspension systems are mainly formulated with dihydrolevoglucosenone as a cellulose derived non-toxic solvent, and cellulose acetate butyrate as a polymer-assisted binder/agent according to the formulation proposed by Pan et al. [7]. The solvent was selected based on its eco-compatibility and biodegradability, and also because it can provide higher concentration of graphene ink, as showed in the same work [7]. The conductive loading filler is made of High Reactivity Carbonaceous Material (HRCM) which can be easily produced in large quantities and shows comparable sheet resistance values to other graphene-based particles in ink suspensions. The combined effect of filler concentration and applied shear rate is investigated in terms of shear viscosity response as a function of temperature. The non-Newtonian flow of shear flow ramps at constant temperature are reported to depend on both HRCM load and the testing temperature. Moreover, temperature ramps at a constant shear rate reveal a different viscosity–temperature dependence from what is observed in shear flow ramps while maintaining the same filler concentration. An apparent departure from the well-known Vogel–Fulcher–Tamman (VFT) relationship as a function of the applied shear rate is also reported. Furthermore, suspensions are used to produce ink strips to test their electrical resistance properties and to validate their usability in view of possible applications, showing that it is possible to fit suitable ranges of sheet resistance. However, our rheological findings are partially in contrast to results reported so far for several carbonaceous-based suspensions [19,20,32].

## 2. Materials and Methods

### 2.1. Materials

**Figure 1:**
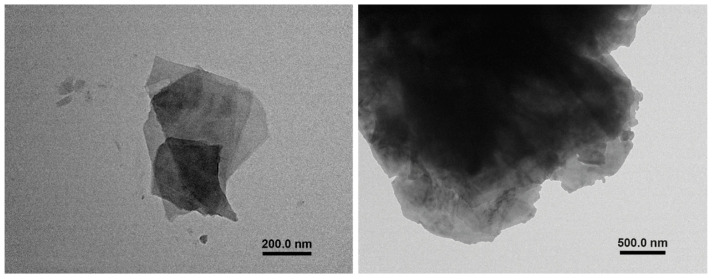
TEM images of HRCM powder after ultrasonication in isopropanol at two different length scales, showing the presence of superimposed high-ratio flakes with few layers of graphene sheets at the micro-sized length scale.

In this work, HRCM was employed as the conductive filler particles of ink suspensions and was provided by Inter Est Group (Cagliari-Italy). HRCM is a powder made of expanded graphite obtained via thermochemical reaction as reported in Patent US 7842271B2, which was previously morphologically characterized by Spissu and coworkers [40,41]. After being dispersed in isopropanol by ultrasonication (GT SONIC-D6, 40 kHz, 150 W) for 4 h, HRCM is present mainly in the form of superimposed high-ratio flakes with few layers of graphene sheets, as shown by transmission electron microscopy (TEM) images in Figure 1. The binder polymer and the solvent medium are, respectively, cellulose acetate butyrate, CAB (average molecular weight M_w_ = 70 kg/mol, Sigma Aldrich), and dihydrolevoglucosenone (≥98.5% purity, average molecular weight M_w_ = 128.13 g/mol, boiling temperature T_b_ = 227 °C, Sigma Aldrich, St. Louis, MO, USA) also known under the commercial name Cyrene. All the aforementioned materials were used as received from the manufacturer.

### 2.2. Conductive Ink Suspension Preparation

For the preparation of the conductive ink suspensions, HRCM and binder polymer with initial HRCM concentrations of 0.08, 0.16, 0.40, and 0.80 wt% and a constant particle–binder weight ratio of 10:1 were embedded in the solvent medium (Cyrene) after being weighed. The dispersion of filler particles and binder into the solvent medium was achieved by degassing sonication for eight hours in an ultrasound bath (Xmoonant Ultrasonic Cleaner, 40 kHz, 35 W) to obtain a homogeneous suspension with good electrical conductivity properties according to the results reported by Pan et al. [7]. In order to achieve suspensions at the concentration of 2.40 wt%, the sonicated suspension at 0.80 wt% was concentrated up to 2.40 wt% via static evaporation on a lab-desk hotplate under the hood and gentle stirring at 230 °C (near the boiling temperature of the solvent). Before any sample loading and testing, the suspension was gently stirred with a magnetic stirrer at the constant rotational speed of 100 1/min for ten minutes. All the described sample-preparation steps were carried out at room temperature unless otherwise stated.

### 2.3. Conductive Strip Preparation

The ink dispersions at all prepared HRCM concentrations were used for the preparation of 12 cm long and 1 cm wide strips on cardboard. The strips were treated at 100 °C for 5 days before any measurement. The two lower concentrations, characterized by a lower viscosity, were spread with a brush, while the higher-viscosity inks were spread over an acetate mask. This selection was made accordingly to viscosity, as inks with the lowest viscosity were unsuitable for the acetate mask and, on the other hand, spreading inks with the highest viscosity by brush in a homogeneous way was unfeasible. Subsequently, the ink strips were pressed between two hot plates twice for 10 s at 300 °C. The samples’ sheet resistance (Rs) was measured before and after the latter heat treatment (HT), as described in the following Section 2.4.

### 2.4. Static Electrical Conductivity

The Rs of the strips was measured after each thermic treatment through two 2 mm flat metal contacts spaced 10 cm apart and connected to a Tonghui TH2826 LCRmeter. Data were collected in DCR mode after calibration before every set of measurements. Reported values are the means of five measurements.

### 2.5. Steady Shear Rheology

Steady simple shear measurements were carried out on conductive ink suspensions using an MCR 102 rheometer (Anton Paar) in strain-controlled mode equipped with a Peltier temperature control system having a resolution of ±0.1 °C and a Peltier hood as solvent trap connected to an external bath circulator in order to guarantee a uniform temperature gradient within the tested sample. Stainless steel parallel plates with 50 mm and 25 mm diameters were used as measuring fixtures while setting the gap between plates equal to 1 mm as the sample loading position. All the measurements were performed on the fresh sample which was loaded at room temperature with the help of a metallic spatula. In order to avoid sample under-filling issues, proper sample loading was always verified by eye-check preliminary testing. A small amount of low-viscosity silicone oil (viscosity = 49.3 mPa · s at 25 °C, Alfa Aesar) was added at the free edges of the sample to minimize solvent evaporation during measurements of twenty minutes and longer.

Steady shear viscosity measurements were performed at temperatures from 5 to 70 °C by applying shear rates $\dot{\gamma }\;$ between 0.01 and 1000 s^−1^ as both single constant values and as a log ramp sampling rate. Furthermore, steady temperature ramp measurements were carried out by applying temperature values from 5 to 80 °C with a heating rate of 1 °C/min. All the aforementioned measures were performed with an initial delay time of five minutes at the set-up temperature in order to achieve thermal equilibrium of the sample after its loading. 

## 3. Results

### 3.1. Static Electrical Conductivity

Table 1 reports the Rs values of the ink strips before and after the HT for the selected concentrations. As expected, a lower sheet resistance is observed for more concentrated formulation of the ink suspension. In fact, after the annealing process, the sample at 0.08 wt% shows a sheet resistance around 350 Ω·sq^−1^ and the sample at 2.40 wt% a very low Rs (12 Ω·sq^−1^). The similar observed values for samples at 0.40 and 0.80 wt% highlight the importance of the spreading method on the properties of the ink, because brushing can allow one to obtain a higher thickness.

The comparison between the Rs values before and after the HT shows the effect of the second thermic treatment at higher temperatures, even for a very short period of time. In fact, in all cases, a consistent decrease in the sheet resistance was observed, which was more than halved after the treatment. With the aim of comparison of some reference values of related graphene-based conductive inks, Majee et al. formulated a highly stable exfoliated graphene-based ink as a more accessible alternative to ITO transparent films [39]. Such formulation reached 826 Ω·sq^−1^ as printed and 260 Ω·sq^−1^ after annealing treatment. The general optoelectronic properties make it a valid candidate for applications such as thin transparent conductive films for touch panels. Li and coworkers synthesized a very stable conductive ink based on reduced graphene oxide and Ag@Au triangular nanoplates with a sheet resistance around 150 Ω·sq^−1^ [42]. The low resistance and other optical properties make it a good option for integrated circuits and highly transparent devices. Deng et al. reported the preparation of a highly stable graphene conductive ink decorated with silver nanoparticles characterized by a sheet resistance of 20 Ω·sq^−1^ after a 30-minute annealing at 400 °C, which made it suitable for printed flexible electronics [43]. Moreover, the authors interestingly highlighted the importance of the annealing process: in fact, performing the annealing at 250 °C led to an ink with a higher sheet resistance (180 Ω·sq^−1^).

### 3.2. Steady Shear Viscosity

#### 3.2.1. Constant Temperature Flow Curves

**Figure 2:**
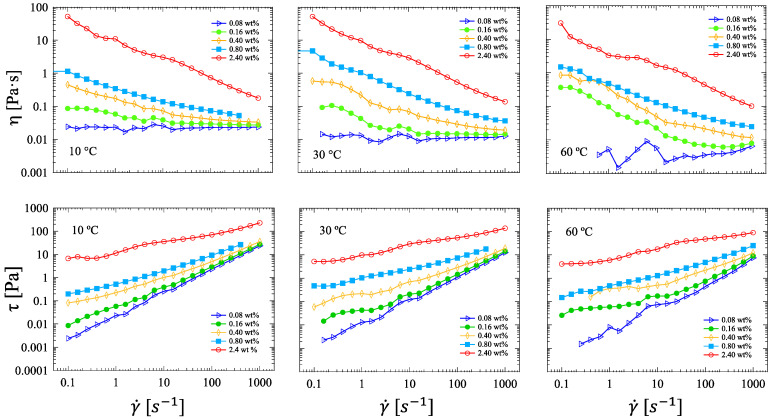
Flow curves in terms of both shear viscosity (upper line) and shear stress (bottom line) of ink suspensions at five different HRCM concentrations obtained via log ramp sampling rate at three temperatures.

Preliminary steady shear viscosity tests on the ink suspension at an HRCM concentration of 0.80 wt% showed a remarked non-Newtonian flow behavior with shear viscosity values of two orders of magnitude greater than the pure solvent and the solvent–binder mixture, which in turn are Newtonian fluids (Figure S1). Therefore, one can conclude that the observed non-Newtonian flow behavior of the ink is mostly related to the presence of HRCM at the filler:polymer ratio 10:1 used in this work. Figure 2 shows flow curves in terms of both shear viscosity (upper line) and shear stress (bottom line) of ink suspensions at four different HRCM concentrations obtained via log ramp sampling rate at three temperatures. As generally expected, an increase in shear viscosity and a transition in the flow behavior from Newtonian fluid (at 0.08 wt% only) to shear-thinning or pseudoplastic fluid upon increasing filler concentration was observed. Moreover, the flow transition mentioned above was shown to be affected by temperature as well: The higher the temperature, the more apparent the non-Newtonian flow behavior at the same filler content. However, by increasing HRCM concentration 30 times (up to 2.40 wt%), the temperature effect on flow transition was negligible. It is worth noting that depending on the HRCM content, the shear viscosity may increase and decrease with temperature. The assessment of a more detailed viscosity–temperature dependence in shear flow is then mandatory.

**Figure 3:**
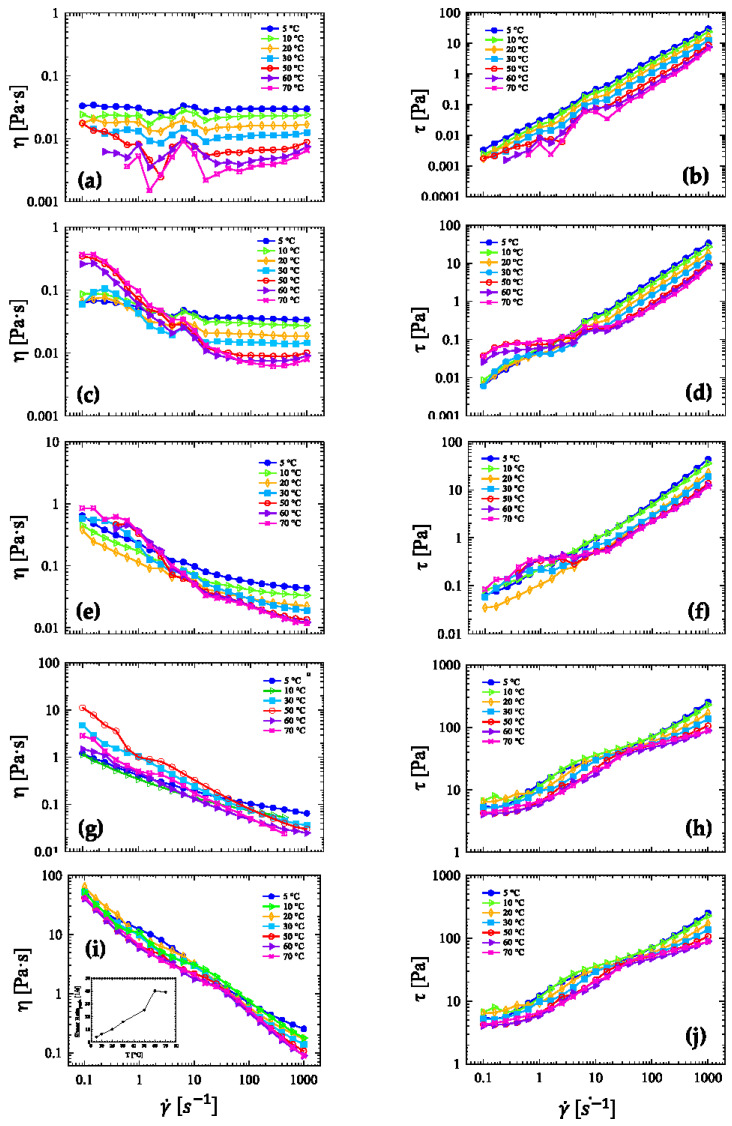
Flow curves in terms of shear viscosity and shear stress of HRCM-based suspensions with: (**a**,**b**) 0.08, (**c**,**d**) 0.16, (**e**,**f**) 0.40, (**g**,**h**) 0.80, and (**i**,**j**) 2.40 wt% HRCM load. Data were collected at constant temperature ranging from 5 to 70 °C via log ramp sampling rate. Inset in (**i**): Shear rate at the observed shear viscosity peak as a function of temperature.

Figure 3 presents flow curve data in terms of shear viscosity and shear stress at different temperatures ranging from 5 to 70 °C for HRCM-based ink suspensions at 0.08, 0.16, 0.40, 0.80, and 2.40 wt% HRCM concentration. At 0.08 wt% HRCM the ink showed a Newtonian fluid response at all applied shear rates and temperatures up to 30 °C while decreasing shear viscosity and stress upon increasing temperature where HRCM particles did not show a preference in their orientation with respect to the flow direction (Figure 3a,b). However, starting from 50 °C, a peculiar incipit of shear thickening was observed mainly in the last decade of applied shear rates (100 to 1000 s^−1^). At higher HRCM contents, a transition from Newtonian to non-Newtonian flow with a shift in the temperature dependence of viscosity became more and more apparent but in a different way depending on the ink concentration. For inks with 0.16 wt% HRCM the Newtonian region at high shear rates (10 to 1000 s^−1^) was preceded by shear-thinning flow at mid shear rates (1 to 10 s^−1^), and a quasi-Newtonian region at small rates (below 1 s^−1^). The shear viscosity was seen to increase and decrease with increasing temperature at low and high applied shear rates, for the viscosity and the shear stress, respectively (Figure 3c,d). To note, the presence of small shear thickening at high shear rates was again observed and appeared at 70 °C only. The overall response leads us to speculate that temperature increase may induce a more complex aggregation state of HRCM particles that tends to be oriented under shear flow and starts jamming after a certain shear rate value. At 0.40 wt% HRCM the Newtonian flow at low shear rates was no longer observed, and shear-thinning followed by high-rate Newtonian flow was the main non-Newtonian feature with shear viscosity and shear stress always decreasing when temperature was increased up to 20 °C (Figure 3e,f). At a temperature higher than 20 °C and shear rates lower than 5 s^−1^, the increase in both shear viscosity and stress and increment of the shear thinning stretch was observed again. This latter aspect was even more emphasized in suspensions at 0.80 wt% HRCM where increasing temperature from 5 to 50 °C induced both shear viscosity and shear stress increase, having a more pseudoplastic character starting at ~200 s^−1^ and below (Figure 3g,h). On the other hand, further temperature increments induced a drop in viscosity and stress values along the whole spectrum of rates. Conversely, suspensions with the highest wt% HRCM content always showed shear viscosity and shear stress decrease when increasing temperature at all applied shear rates, as previously seen for the 0.08 wt% HRCM ink (Figure 3i,j). However, the highly concentrated HRCM-based ink showed a mixed non-Newtonian flow response having two apparent shear-thinning regions: the transition from one shear-thinning region to the other was set by a critical value of the applied shear rate, which in turn depended on the testing temperature (Figure 3i Inset). The standard deviation for some flow curve replicates was calculated, and the results are reported in the Supplementary Materials (Table S1).

**Figure 4:**
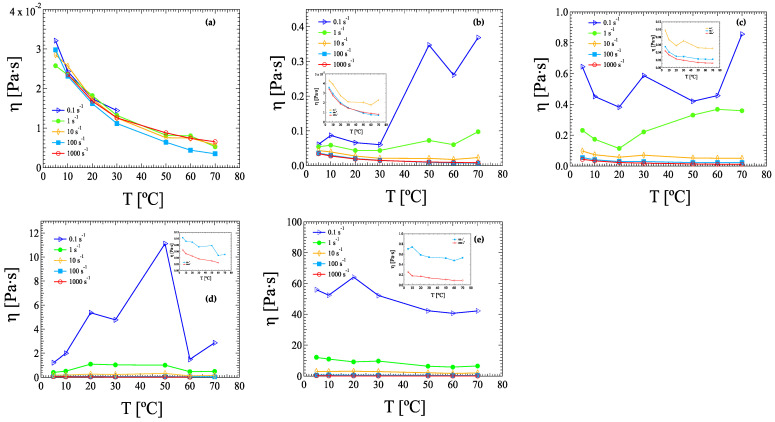
Viscosity dependence on temperature of inks and pastes with HRCM loads of: (**a**) 0.08, (**b**) 0.16, (**c**) 0.40, (**d**) 0.80, and (**e**) 2.40 wt%. Data are extracted from flow curve measurements carried out at constant temperature ranging from 5 to 70 °C via log ramp sampling rate reported in Figure 3.

Figure 4 shows the shear viscosity dependence on temperature for ink suspensions with different HRCM loads. Data were extracted from the flow curve measurements carried out at constant temperature, shown in Figure 3. Except for the lowest HRCM load case (Figure 4a), all the filled suspensions presented a shear-rate-dependent viscosity–temperature relationship. From 0.16 to 0.80 wt% HRCM and from high to low applied shear rate, viscosity values went from decreasing to increasing with temperature increase, followed by a sudden decrease at the lowest applied rate (0.1 s^−1^) for 0.80 wt% only (Figure 4d). At 2.40 wt% HRCM shear viscosity always increased with temperature at all applied shear rates, except for 1000 s^−1^ where temperature independence was observed (Figure 4e and Inset). It is worth noting that an exponential-like viscosity–temperature relationship was observed for the lowest HRCM load, i.e., 0.08 wt%, at all the applied rates (Figure 4a) and also for an HRCM load of 0.16 wt% at shear rates of 100 and 1000 s^−1^ only (inset of Figure 4b). In these two cases, the viscosity-temperature relationship was shown to obey the Vogel–Fulcher–Tamman (VFT) equation [44]. More details are reported in the Discussion section. Departure from exponential-like shear viscosity decrease with temperature was observed for all the other HRCM-filled ink suspensions.

#### 3.2.2. Constant Shear Rate Temperature Ramps

**Figure 5:**
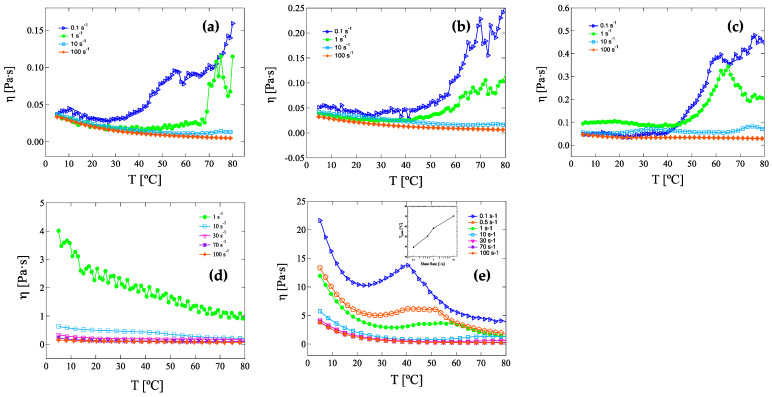
Steady temperature ramp measurements at 1 °C/min heating rate on inks and pastes with HRCM loads of (**a**) 0.08, (**b**) 0.16, (**c**) 0.40, (**d**) 0.80, and (**e**) 2.40 wt%. Inset of Figure 5e: Temperature at the observed shear viscosity peak as a function of the applied shear rate.

Direct assessment of the viscosity–temperature dependence on the applied shear rate in HRCM-based suspensions was carried out via steady temperature ramp measurements at 1 °C/min heating rate while imposing a constant shear rate on suspensions at the same HRCM load (Figure 5). From 0.08 to 0.40 wt% HRCM, shear viscosity values showed either a weak decrease as a function of temperature in the region of applied shear rates between 100 and 10 s^−1^ (Figure 5a,b) or temperature independence in the same shear rate range (Figure 5c). By further decreasing the value of the applied shear rate from 10 to 0.1 s^−1^, a progressive increase in viscosity started at a temperature value which was found to decrease as the applied shear rate decreased. The measured shear viscosity values at the highest temperature were around three to five times higher than values at the lowest temperature. By increasing ten times the initial concentration of HRCM load in the ink suspension, i.e., 0.80 wt%, an almost continuous decrement in shear viscosity as a function of temperature at the lowest shear rate (1 s^−1^) was shown, then it flattened for higher shear rate values, and eventually became independent of temperature for the highest shear rate values, from 30 to 100 s^−1^ (Figure 5d). On the other hand, by increasing 30 times the initial ink concentration of HRCM, the temperature dependency of shear viscosity became more complex, showing an even stronger shear-rate–temperature interdependence with shear viscosity. After an initial decrease with temperature increase, shear viscosity showed a temperature-driven peak increase which appeared at higher and higher temperatures as the applied shear rate increased, before starting to decrease again (Figure 5e). Noteworthy, at this HRCM load, the temperature of the first peak showed a power law dependence on the applied shear rate (Figure 5e Inset). At the highest shear rate of 100 s^−1^, on the contrary, shear viscosity continuously decreased upon reaching a plateau value at high temperatures. The standard deviation for some steady temperature ramp replicates was calculated and the results are reported in the Supplementary Materials (Table S1). As previously observed for the rate-ramp data sets at a fixed temperature, the viscosity–temperature relationship of temperature-ramp data sets at a fixed shear rate was seen to obey the VFT equation at 100 s^−1^ and 0.08 wt% and 0.16 wt% HRCM. Fitting parameters and standard deviations are reported in Table A1 and Table A2 in Appendix A.

## 4. Discussion

The shear flow behavior of HRCM-filled inks and pastes and the temperature-related variations in viscosity are discussed in the following, considering the role of the applied strain history in the temperature domain.

The addition of HRCM particles into a Newtonian solvent base induces a transition from Newtonian to non-Newtonian flow, whose features depend on the applied strain history and the testing temperature. In the case of shear strain rate applied following a logarithmic ramp at a constant temperature, for a given filler content, the temperature dependence of viscosity shifts from constant decreasing at high shear rates to quasi-constant increasing at low shear rates within 65 °C step, except for the suspensions with the lowest and the highest HRCM loads (Figure 3). This is in contrast to previous published works where suspensions filled with various carbonaceous particles are reported to show a viscosity–temperature relation of quasi-constant decrease independent of filler concentration and applied rate [25,26]. The cited literature results are supported by the finding that temperature-related variations in viscosity observed for the filled suspensions are very similar to those of the base solvent. On the other hand, our results may describe a temperature-related mechanism of internal resistance increase in the base solvent by filler particles superposed to that induced by solely the dispersion of filler particles as recently reported for carbonaceous-filled suspensions [25,32,45].

Furthermore, the onset and the type of non-Newtonian flow are also seen to depend on both the HRCM load and the testing temperature. Three empirical viscoelastic models for modelling the non-Newtonian behavior, i.e., the Carreau–Yasuda model [46], the modified Ostwald–de Waele model by Sisko [47], and the Herschel–Bulkley model [48], are used to interpret the shear-rate dependence of shear viscosity as a function of both HRCM load and temperature shown in Figure 3. Equation formulas, fitting parameters along with their confidential intervals (CI), and the mean squared displacement (MSE) as a metric of the fit goodness of the aforementioned viscoelastic models are reported in detail in Appendix B for ink suspensions filled with 0.16, 0.40, and 0.80 wt% HRCM in Table A3, Table A4, Table A5, Table A6 and Table A7, respectively.

**Figure 6:**
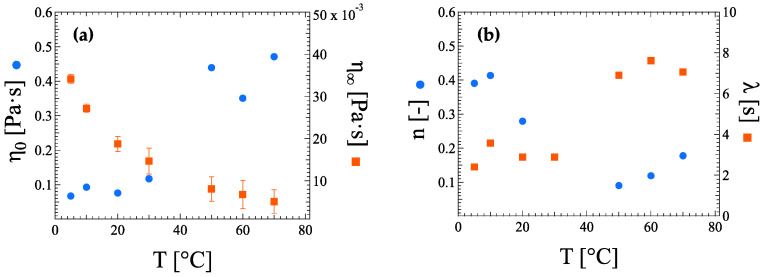
Carreau–Yasuda model fitting parameters with error bars of (**a**) asymptotic viscosities at zero *η*_0_ and infinity *η_∞_* shear rates, and (**b**) flow behavior, or power, index *n* and flow consistency *λ* as a function of temperature for the ink suspension at 0.16 wt% HRCM content.

The Carreau–Yasuda model was applied exclusively to ink suspensions with 0.16 wt% HRCM load (Table A3). The flow curve shows indeed a non-Newtonian transient shear thinning flow region that can be described by means of a power law and is delimited by both low-rate and high-rate Newtonian plateaus which in turn are defined by the zero-shear viscosity *η*_0_ and the infinity-shear viscosity *η_∞_*, respectively. As already seen for other carbonaceous filler load increases [25], the zero-shear viscosity *η*_0_ and the infinity-shear viscosity *η_∞_* increases and decreases, respectively, with increasing temperature (Figure 6a). On the other hand, the flow consistency index *λ* (i.e., 1/${\dot{\gamma }}_{cr}$ at which the onset of transient shear thinning is observed) and flow behavior, or power, index *n* unveil a discontinuous shear-rate dependence of viscosity towards shear-thinning pseudoplasticity at a fixed filler load with increasing temperature (Figure 6b). More specifically, a sudden increase and decrease in the temperature dependence of the parameters *n* and *λ*, respectively, are observed at a temperature higher than 30 °C.

**Figure 7:**
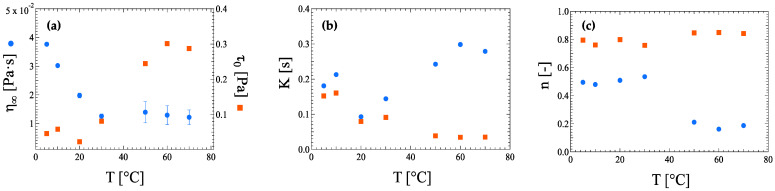
Comparison between Sisko equation (circle) and Herschel–Bulkley model (square) fitting parameters with error bars of (**a**) infinity-shear viscosity *η_∞_* and zero-shear stress *τ*_0_, (**b**) flow consistency index *K*, and (**c**) flow behavior index *n* for ink suspensions with 0.40 wt% HRCM load.

At 0.40 wt% HRCM the low-rate Newtonian plateau is no longer appreciated; thus the Sisko equation has been tested as an approximation of the empirical Carreau–Yasuda equation taking into account the shear-thinning region and the high-rate Newtonian plateau only by means of the flow consistency *K* and power *n* index, and the infinity viscosity *η_∞_*, respectively (Table A4). Here, the index *K* has the same physical meaning as previously seen for the Carreau–Yasuda model but marked with a different letter to better identify the type of model used. As already observed for ink suspensions at a lower HRCM load, the pseudoplasticity of the 0.40 wt% HRCM ink suspensions shows a pivotal change at a temperature of around 30 °C. In particular, the infinity viscosity *η_∞_* decreases with increasing temperature up to 30 °C, then it reaches a plateau value (Figure 7a), whereas the *n* and *K* parameters describing the degree of deviation from Newtonianity and its onset, respectively, show temperature quasi-independence with a sudden decrease and increase for *n* and *K* values, respectively, at 30 °C (Figure 7b,c). The degree of Newtonian deviation shows typical values for shear thinning fluids, i.e., 0.2 < *n* < 0.8, then it suddenly decreases to values lower than 0.2 at higher temperatures indicating a non-Newtonian flow transition towards yielding-like fluids. In this light, the Herschel–Bulkley model and its accuracy were tested as well on the same HRCM-filled ink where a new parameter, the zero-shear, or yield, stress *τ*_0_, is introduced (Table A5). Compared to the results previously reported for the Sisko equation, the pseudoplasticity temperature dependence of the suspension is reported to be different. With increasing temperature, the zero-shear stress *τ*_0_ presents a step-like increase at 30 °C (Figure 7a) whereas the *K* parameter slowly decreases upon reaching a plateau value (Figure 7b), and the *n* parameter oscillates around a constant value of 0.8 (Figure 7c). This indicates a strengthening of the fluid yielding feature at low shear rates on one hand, and an almost constant shear-thinning behavior with reduced shear rate interval on the other, when temperature is raised.

**Figure 8:**
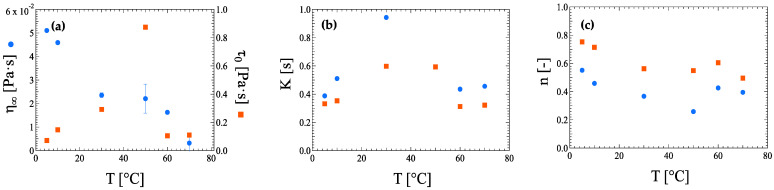
Comparison between Sisko equation (circle) and Herschel–Bulkley model (square) fitting parameters with error bars of (**a**) infinity-shear viscosity *η_∞_* and zero-shear stress *τ*_0_, (**b**) flow consistency index *K*, and (**c**) flow behavior index *n* for ink suspensions with 0.80 wt% HRCM load.

Both models, the Sisko and Herschel–Bulkley equations, were used to fit flow curve data as a function of the temperature of ink suspensions with 0.80 wt% HRCM load as well (Appendix B—Table A6 and Table A7). The comparison of the two models is shown in Figure 8. The infinity viscosity *η_∞_* decreases quasi-monotonically with temperature increase, whereas the zero-shear stress *τ*_0_ increases up to 50 °C, then suddenly drops to a plateau value. The *n* and *K* parameters for pseudoplasticity have very similar qualitative temperature dependence with increasing temperature among the two models: The *K* parameter shows similar values at both low and high temperatures while increasing at intermediate temperatures (Figure 8b), and the *n* parameter decreases down to a plateau value starting at temperatures higher than 30 °C (Figure 8c). Although the Sisko equation and the Herschel–Bulkley model try to describe the pseudoplasticity of a fluid in a different way, it is noteworthy that both models show comparable MSE for ink suspensions at 0.40 and 0.80 wt% HRCM load (Appendix B—Table A4, Table A5, Table A6 and Table A7). This makes it difficult to choose one model over the other. A more feasible suggestion could be to test both models and take into account the parameters which describe the most interested flow region.

In regard to the temperature-related variations in dynamic shear viscosity, different temperature dependence as a function of HRCM load and applied shear rate have been reported in this work (Figure 4 and Figure 5). In particular, at a fixed filler concentration, results differ according to the strain–temperature history set to the ink suspension. Hamze and coworkers report a decrease in dynamic viscosity with increasing temperature for seventeen graphene-based suspensions at eight different temperatures for filler loads from 0.1 to 0.5 wt% [49]. This viscosity behavior of the filled suspensions retraces that of the base fluids, i.e., the universal viscosity–temperature dependence of liquids [50], except for one system only. Similar observations were reported by Vallejo et al. for six carbonaceous-filled suspensions with filler loads up to 2.0 wt% [26]. The departure of our viscosity–temperature dependence from previously reported works can be partially explained by taking into account the likely instability of filled suspensions under shear at increasing temperature for a long period of time, as pointed out by Hamze et al. [49]. However, it is noteworthy that, for our HRCM-filled suspensions, the interplay among temperature, filler concentration, and applied shear rate gives a non-trivial viscosity–temperature relationship greatly departing from that of liquids. On the other hand, ink suspensions at 0.08 and 0.16 wt% HRCM loads show the universal viscosity decrease with increasing temperature for liquids mainly at 100 to 1000 s^−1^ applied shear rate regardless of the shear–temperature protocol. Moreover, fitting results of viscosity as a function of temperature from flow curves and temperature ramps at 100 s^−1^ for the VFT equation show very similar values among the two experimental protocols and to those obtained for the base solvent (Appendix A—Table A1 and Table A2).

## 5. Conclusions

In the present study, a shear rheological characterization in the temperature domain of low-cost, production-scalable, and environmentally sustainable conductive HRCM-based ink suspensions is reported. Preliminary measurements of static electrical conductivity show values of sheet resistance of HRCM particles after ink deposition on a substrate and a short high temperature treatment in the range of 5 to 136 Ω·sq^−1^ depending on the conductive filler concentration and deposition method. The investigated ink is then a promising material for certain technical applications such as thin-film transistors and transparent electrodes. The characterization of ink suspensions in shear flow reveals a non-trivial temperature dependence of dynamic viscosity, highly departing from the more universal solvent medium response. In particular, shear flow protocols at fixed and variable temperatures show similar or different temperature dependence at the same HRCM concentration and applied shear rate highlighting the interplay role of all of the three variables (i.e., temperature, filler concentration, and shear rate). Furthermore, the well-known VFT relationship for viscosity–temperature dependence is reported to hold in dilute conditions only (i.e., 0.08 wt% HRCM load), proving this concentration value to be the threshold limit at and below which the VFT relationship applies. Our finding differs from previously reported results.

## Figures and Tables

**Table 1:** Sheet resistance values before and after HT for four concentrations of the ink formulation.

Concentration *(*wt%)	Spreading Method	Pre-HT R_s_ *(*Ω·sq^−1^*)*	Post-HT R_s_ *(*Ω·sq^−1^*)*
0.08	Brush	347 ± 15	136 ± 8
0.40	Brush	115 ± 2	52 ± 7
0.80	Spatula	108 ± 2	37 ± 1
2.40	Spatula	12 ± 1.8	5.2 ± 0.3

## Data Availability

Not applicable.

## Supplementary Materials

The following supporting information can be downloaded at: https://www.mdpi.com/article/10.3390/nano13010021/s1, Figure S1: Flow curve comparison between 0.80 wt% HRCM ink and its components; Table S1: Computation of the standard deviation for some replicates of flow curve and temperature ramp measurements.

## Appendix A. Vogel–Fulcher–Tamman Equation Parameter Fitting

**Table A1:** Fitting parameters (*A*, *B*, *T*_0_), confidence intervals (CI), and mean square error (MSE) values as a function of HRCM load obtained from the Vogel–Fulcher–Tamman equation [44], modeling the 5 to 70 °C temperature range for flow curve data sets of solvent medium and HRCM-based ink suspensions.

Vogel–Fulcher–Tamman Equation— $\eta =exp{\left(A+\frac{B}{T-T_{0}}\right)}_{\;}$							
HRCM Load	$A\;{\left[Pa\cdot s\right]}^{\;}$	CI	*B*	CI	*T*_0_ [K] *^a^*	CI	MSE
0 wt%	−9.18	0.27	674.02	77.30	159.08	7.98	3.46 × 10^−8^
0.08 wt%	−10.65	4.41	1123.31	1551.98	120.48	120.88	1.06 × 10^−7^
0.16 wt%	−7.38	1.02	367.61	223.78	187.54	32.71	7.46 × 10^−8^

*^a^* Infinity-viscosity temperature.

**Table A2:** Fitting parameters (*A*, *B*, *T*_0_), confidence intervals (CI), and mean square error (MSE) values as a function of HRCM load obtained from the Vogel–Fulcher–Tamman equation [44], modeling the 5 to 70 °C temperature range for steady temperature ramp data sets of solvent medium and HRCM-based ink suspensions.

Vogel–Fulcher–Tamman Equation— $\eta =exp{\left(A+\frac{B}{T-T_{0}}\right)}_{\;}$							
HRCM Load	*A*	CI	*B*	CI	*T*_0_ *^a^*	CI	MSE
0 wt%	−9.18	0.27	674.02	77.30	159.08	7.98	3.46 × ^10−8^
0.08 wt%	−9.61	0.33	1020.51	124.94	114.58	11.39	3.39 × ^10−8^
0.16 wt%	−8.78	0.24	906.18	95.33	109.58	10.13	3.39 × ^10−9^

*^a^* Infinity-viscosity temperature.

## Appendix B. Non-Newtonian Viscoelastic Model Parameter Fitting

**Table A3:** Fitting parameters (*η_0_*, *η_∞_*, *λ*, *n*), confidence intervals (CI), and mean square error (MSE) values as a function of temperature obtained from the Carreau–Yasuda model [46] modeling the 0.1 to 1000 s^−1^ shear rate range for flow curve data sets of ink suspensions with a 0.16 wt% HRCM load.

Carreau–Yasuda Model— $\frac{\eta -\eta_{\infty }}{\eta_{0}-\eta_{\infty }}={\left[1+{\left(\lambda \dot{\gamma }\right)}^{a}\right]}^{\left(n-1\right)/a^{*}}$									
Temperature (°C)	$\eta_{0}\;\left(Pa\cdot s\right)$ *^a^*	CI	$\eta_{\infty }\;\left(Pa\cdot s\right)$ *^b^*	CI	*λ (s) ^c^*	CI	*n ^d^*	CI	MSE
5	0.067143	0.0079	0.034206	0.0031	2.39764	3.0496	0.390552	0.447	1.03 × 10^−3^
10	0.092785	0.0133	0.02717	0.0026	3.571147	3.0108	0.413467	0.2128	1.03 × 10^−3^
20	0.07624	0.0128	0.018753	0.002	2.883527	2.4633	0.279065	0.2952	1.80 × 10^−3^
50	0.439401	0.1712	0.008091	0.0013	6.905023	4.8826	0.090287	0.13	0.002906
60	0.35062	0.1633	0.00678	0.0012	7.616378	6.4145	0.118849	0.1382	0.003355
70	0.471002	0.1773	0.005096	0.0011	7.054768	4.9611	0.177683	0.0967	0.002782

*^a^* Zero-shear viscosity; *^b^* Infinity-shear viscosity; *^c^* Flow consistency index; *^d^* Flow behavior index; * *a* = 2.

**Table A4:** Fitting parameters (*η_∞_*, *K*, *n*), confidence intervals (CI), and mean square error (MSE) values as a function of temperature obtained from the modified Ostwald–de Waele model by Sisko, or Sisko equation [47], modeling the 0.1 to 1000 s^−1^ shear rate range for flow curve data sets of ink suspensions with a 0.40 wt% HRCM load.

Sisko Equation– $\eta =\eta_{\infty }+K{\dot{\gamma }}^{n-1}$							
Temperature (°C)	$\eta_{\infty }\;\left(Pa\cdot s\right)$ *^a^*	CI	$K\;\left(Pa\cdot s^{n}\right)$ *^b^*	CI	*n ^c^*	CI	MSE
5	0.037657	0.0025	0.180791	0.0065	0.495193	0.0226	2.58 × 10^−4^
10	0.030213	0.0032	0.212824	0.0099	0.479635	0.0295	5.66 × 10^−4^
20	0.019788	0.0024	0.092808	0.006	0.5090	0.0405	8.10 × 10^−4^
30	0.012586	0.0039	0.14401	0.0159	0.535122	0.076	7.44 × 10^−4^
50	0.013952	0.0032	0.242429	0.0685	0.211372	0.1662	0.003695
60	0.012935	0.0027	0.298512	0.0773	0.162102	0.1464	3.28 × 10^−3^
70	0.0122	0.0024	0.278803	0.0633	0.187128	0.1274	0.002569

*^a^* Infinity-shear viscosity; *^b^* Flow consistency index; *^c^* Flow behavior index.

**Table A5:** Fitting parameters (*τ*_0_, *K*, *n*), confidence intervals (CI), and mean square error (MSE) values as a function of temperature obtained from the Hershel–Bulkley model [48], modeling the 0.1 to 1000 s^−1^ shear rate range for flow curve data sets of ink suspensions with a 0.40 wt% HRCM load.

Hershel–Bulkley Model— $\tau =\tau_{0}+K{\dot{\gamma }}^{n}$							
Temperature (°C)	$\tau_{0}\;\left(Pa\cdot s\right)$ *^a^*	CI	$K\;\left(Pa\cdot s^{n}\right)$ *^b^*	CI	*n ^c^*	CI	MSE
5	0.04605	0.0125	0.152182	0.0203	0.794973	0.03	1.83 × 10^−3^
10	0.058292	0.012	0.160333	0.0181	0.761024	0.025	1.17 × 10^−3^
20	0.022862	0.0058	0.0793	0.0096	0.799159	0.0273	1.54 × 10^−3^
30	0.080961	0.0522	0.091285	0.0241	0.757823	0.0495	0.001267
50	0.24454	0.0629	0.038598	0.018	0.847034	0.0833	0.002266
60	0.301386	0.062	0.034712	0.0161	0.849241	0.0819	0.001871
70	0.287441	0.0539	0.034984	0.0144	0.842573	0.0726	0.001488

*^a^* Zero-shear stress; *^b^* Flow consistency index; *^c^* Flow behavior index.

**Table A6:** Fitting parameters (*η_∞_*, *K*, *n*), confidence intervals (CI), and mean square error (MSE) values as a function of temperature obtained from the modified Ostwald–de Waele model by Sisko, or Sisko equation [47], modeling the 0.1 to 1000 s^−1^ shear rate range for flow curve data sets of ink suspensions with a 0.80 wt% HRCM load.

Sisko Equation— $\eta =\eta_{\infty }+K{\dot{\gamma }}^{n-1}$							
Temperature (°C)	$\eta_{\infty }\;\left(Pa\cdot s\right)$ *^a^*	CI	$K\;\left(Pa\cdot s^{n}\right)$ *^b^*	CI	*n ^c^*	CI	MSE
5	0.051078	0.0047	0.387735	0.0115	0.551372	0.0182	2.16 × 10^−4^
10	0.045909	0.0047	0.510981	0.0179	0.458828	0.0221	4.15 × 10^−4^
30	0.023546	0.0048	0.943615	0.043	0.367697	0.0259	9.85 × 10^−4^
50	0.0221	0.01	1.5899	0.1766	0.2587	0.0593	0.006176
60	0.0163	0.0024	0.4358	0.0143	0.4264	0.0195	4.76 × 10^−4^
70	0.003212	0.0037	0.455981	0.0394	0.395258	0.045	0.00384

*^a^* Infinity-shear viscosity; *^b^* Flow consistency index; *^c^* Flow behavior index.

**Table A7:** Fitting parameters (*τ*_0_, *K*, *n*), confidence intervals (CI), and mean square error (MSE) values as a function of temperature obtained from the Hershel–Bulkley model [48] modeling the 0.1 to 1000 s^−1^ shear rate range for flow curve data sets of ink suspensions with a 0.80 wt% HRCM load.

Hershel–Bulkley Model— $\tau =\tau_{0}+K{\dot{\gamma }}^{n}$							
Temperature (°C)	$\tau_{0}\;\left(Pa\cdot s\right)$ *^a^*	CI	$K\;\left(Pa\cdot s^{n}\right)$ *^b^*	CI	*n ^c^*	CI	MSE
5	0.071663	0.0116	0.332065	0.0195	0.752879	0.0133	3.72 × 10^−4^
10	0.147659	0.0189	0.353093	0.0266	0.714884	0.0164	4.56 × 10^−4^
30	0.29244	0.1052	0.597545	0.1298	0.562771	0.0444	0.002463
50	0.874338	0.2397	0.592698	0.2334	0.549265	0.0745	0.004059
60	0.10547	0.04	0.312343	0.0543	0.605262	0.0368	0.002073
70	0.109739	0.0609	0.322211	0.0757	0.495378	0.0471	0.002646

*^a^* Zero-shear stress; *^b^* Flow consistency index; *^c^* Flow behavior index.

## References

[1] Selvam C., Solaimalai Raja R., Mohan Lal D., Harish S. (2017). Overall Heat Transfer Coefficient Improvement of an Automobile Radiator with Graphene Based Suspensions. Int. J. Heat Mass Transf..

[2] Qu J., Shang L., Sun Q., Han X., Zhou G. (2022). Photo-Thermal Characteristics of Water-Based Graphene Oxide (GO) Nanofluids at Reverse-Irradiation Conditions with Different Irradiation Angles for High-Efficiency Solar Thermal Energy Harvesting. Renew. Energy.

[3] Dimiev A.M., Eigler S. (2017). Graphene Oxide: Fundamentals and Applications.

[4] Pradhan D., Ghosh S.P., Gartia A., Sahoo K.K., Bose G., Kar J.P. (2020). Modulation of Microstructural and Electrical Properties of Rapid Thermally Synthesized MoS2 Thin Films by the Flow of H_2_ Gas. Superlattices Microstruct.

[5] Zhang H., Fonseca A.F., Cho K. (2014). Tailoring Thermal Transport Property of Graphene through Oxygen Functionalization. J. Phys. Chem. C.

[6] Mortazavi B., Pereira L.F.C., Jiang J.W., Rabczuk T. (2015). Modelling Heat Conduction in Polycrystalline Hexagonal Boron-Nitride Films. Sci. Rep..

[7] Pan K., Fan Y., Leng T., Li J., Xin Z., Zhang J., Hao L., Gallop J., Novoselov K.S., Hu Z. (2018). Sustainable Production of Highly Conductive Multilayer Graphene Ink for Wireless Connectivity and IoT Applications. Nat. Commun..

[8] Helal E., Kurusu R.S., Moghimian N., Gutierrez G., David E., Demarquette N.R. (2019). Correlation between Morphology, Rheological Behavior, and Electrical Behavior of Conductive Cocontinuous LLDPE/EVA Blends Containing Commercial Graphene Nanoplatelets. J. Rheol..

[9] Kazatchkov I.B., Yip F., Hatzikiriakos S.G. (2000). The Effect of Boron Nitride on the Rheology and Processing of Polyolefins. Rheol. Acta.

[10] Lin H.W., Chang C.P., Hwu W.H., Ger M.D. (2008). The Rheological Behaviors of Screen-Printing Pastes. J. Mater. Process. Technol..

[11] Vallejo J.P., Pérez-Tavernier J., Cabaleiro D., Fernández-Seara J., Lugo L. (2018). Potential Heat Transfer Enhancement of Functionalized Graphene Nanoplatelet Dispersions in a Propylene Glycol-Water Mixture. Thermophysical Profile. J. Chem. Thermodyn..

[12] Del Giudice F., Shen A.Q. (2017). Shear Rheology of Graphene Oxide Dispersions. Curr. Opin. Chem. Eng..

[13] Giudice F.D., Cunning B.V., Ruoff R.S., Shen A.Q. (2018). Filling the Gap between Transient and Steady Shear Rheology of Aqueous Graphene Oxide Dispersions. Rheol. Acta.

[14] Choi G.M., Park M., Jeong S.Y., Lee H.S. (2021). Orientation Effect on the Rheology of Graphene Oxide Dispersions in Isotropic Phase, Ordered Isotropic Biphase, and Discotic Phase. J. Rheol..

[15] Ferreira E.H.C., Andrade R.J.E., Fechine G.J.M. (2019). The “Superlubricity State” of Carbonaceous Fillers on Polyethylene-Based Composites in Molten State. Macromolecules.

[16] Li P., Ji L., Li H., Chen L., Liu X., Zhou H., Chen J. (2021). Role of Nanoparticles in Achieving Macroscale Superlubricity of Graphene/Nano-SiO2 Particle Composites. Friction.

[17] Moraes L.R.da.C., Ribeiro H., Cargnin E., Andrade R.J.E., Naccache M.F. (2020). Rheology of Graphene Oxide Suspended in Yield Stress Fluid. J. Nonnewton. Fluid Mech..

[18] Soares Y.C.F., Cargnin E., Naccache M.F., Andrade R.J.E. (2020). Influence of Oxidation Degree of Graphene Oxide on the Shear Rheology of Poly(Ethylene Glycol) Suspensions. Fluids.

[19] Sarsam W.S., Amiri A., Kazi S.N., Badarudin A. (2016). Stability and Thermophysical Properties of Non-Covalently Functionalized Graphene Nanoplatelets Nanofluids. Energy Convers. Manag..

[20] Moghaddam M.B., Goharshadi E.K., Entezari M.H., Nancarrow P. (2013). Preparation, Characterization, and Rheological Properties of Graphene-Glycerol Nanofluids. Chem. Eng. J..

[21] Corker A., Ng H.C.H., Poole R.J., García-Tuñón E. (2019). 3D Printing with 2D Colloids: Designing Rheology Protocols to Predict “printability” of Soft-Materials. Soft Matter.

[22] Arapov K., Rubingh E., Abbel R., Laven J., de With G., Friedrich H. (2016). Conductive Screen Printing Inks by Gelation of Graphene Dispersions. Adv. Funct. Mater..

[23] Vasu K.S., Krishnaswamy R., Sampath S., Sood A.K. (2013). Yield Stress, Thixotropy and Shear Banding in a Dilute Aqueous Suspension of Few Layer Graphene Oxide Platelets. Soft Matter.

[24] Gong S., Chen Q., Moll J.F., Kumar S.K., Colby R.H. (2014). Segmental Dynamics of Polymer Melts with Spherical Nanoparticles. ACS Macro Lett..

[25] Vallejo J.P., Żyła G., Fernández-Seara J., Lugo L. (2018). Rheological Behaviour of Functionalized Graphene Nanoplatelet Nanofluids Based on Water and Propylene Glycol:Water Mixtures. Int. Commun. Heat Mass Transf..

[26] Vallejo J., Żyła G., Fernández-Seara J., Lugo L. (2019). Influence of Six Carbon-Based Nanomaterials on the Rheological Properties of Nanofluids. Nanomaterials.

[27] Murshed S.M.S., Estellé P. (2017). A State of the Art Review on Viscosity of Nanofluids. Renew. Sustain. Energy Rev..

[28] Murshed S.M.S., Santos F.J.V., de Castro C.A.N. (2013). Investigations of Viscosity of Silicone Oil-Based Semiconductor Nanofluids. J. Nanofluids.

[29] Namburu P.K., Kulkarni D.P., Misra D., Das D.K. (2007). Viscosity of Copper Oxide Nanoparticles Dispersed in Ethylene Glycol and Water Mixture. Exp. Therm. Fluid Sci..

[30] Rudyak V.Y., Dimov S.V., Kuznetsov V.V., Bardakhanov S.P. (2013). Measurement of the Viscosity Coefficient of an Ethylene Glycol-Based Nanofluid with Silicon-Dioxide Particles. Dokl. Phys..

[31] Cabaleiro D., Estellé P., Navas H., Desforges A., Vigolo B. (2018). Dynamic Viscosity and Surface Tension of Stable Graphene Oxide and Reduced Graphene Oxide Aqueous Nanofluids. J. Nanofluids.

[32] Hamze S., Cabaleiro D., Maré T., Vigolo B., Estellé P. (2020). Shear Flow Behavior and Dynamic Viscosity of Few-Layer Graphene Nanofluids Based on Propylene Glycol-Water Mixture. J. Mol. Liq..

[33] Kole M., Dey T.K. (2011). Effect of Aggregation on the Viscosity of Copper Oxide–Gear Oil Nanofluids. Int. J. Therm. Sci..

[34] Syam Sundar L., Singh M.K., Sousa A.C.M. (2013). Investigation of Thermal Conductivity and Viscosity of Fe3O4 Nanofluid for Heat Transfer Applications. Int. Commun. Heat Mass Transf..

[35] Fakhari A., Fernandes C., Galindo-Rosales F.J. (2022). Mapping the Volume Transfer of Graphene-Based Inks with the Gravure Printing Process: Influence of Rheology and Printing Parameters. Materials.

[36] Torrisi F., Hasan T., Wu W., Sun Z., Lombardo A., Kulmala T.S., Hsieh G.-W., Jung S., Bonaccorso F., Paul P.J. (2012). Inkjet-Printed Graphene Electronics. ACS Nano.

[37] Pang S., Hernandez Y., Feng X., Müllen K., Pang S., Hernandez Y., Feng X., Müllen K. (2011). Graphene as Transparent Electrode Material for Organic Electronics. Adv. Mater..

[38] De S., Coleman J.N. (2010). Are There Fundamental Limitations on the Sheet Resistance and Transmittance of Thin Graphene Films?. ACS Nano.

[39] Majee S., Song M., Zhang S.L., Zhang Z.B. (2016). Scalable Inkjet Printing of Shear-Exfoliated Graphene Transparent Conductive Films. Carbon.

[40] Petrik V.I. (2010). Mass Production of Carbon Nanostructures. U.S. Patent.

[41] Spissu Y., Barberis A., Bazzu G., D’hallewin G., Rocchitta G., Serra P.A., Marceddu S., Vineis C., Garroni S., Culeddu N. (2021). Functionalization of Screen-Printed Sensors with a High Reactivity Carbonaceous Material for Ascorbic Acid Detection in Fresh-Cut Fruit with Low Vitamin C Content. Chemosensors.

[42] Li L., Gao M., Guo Y., Sun J., Li Y., Li F., Song Y., Li Y. (2017). Transparent Ag@Au–Graphene Patterns with Conductive Stability via Inkjet Printing. J. Mater. Chem. C.

[43] Deng D., Feng S., Shi M., Huang C. (2017). In Situ Preparation of Silver Nanoparticles Decorated Graphene Conductive Ink for Inkjet Printing. J. Mater. Sci. Mater. Electron..

[44] Tammann G., Hesse W. (1926). Die Abhängigkeit Der Viscosität von Der Temperatur Bie Unterkühlten Flüssigkeiten. Z. Für Anorg. Und Allg. Chem..

[45] Bansal N.P., Singh J.P., Ko S.W., Castro R.H.R., Pickrell G., Manjooran N.J., Nair K.M., Singh G. (2013). Processing and Properties of Advanced Ceramics and Composites V.

[46] Bird R.B., Armstrong R.C., Hassager O. (1987). Dynamics of Polymeric Liquids. Volume 1: Fluid Mechanics.

[47] Sisko A.W. (1958). The Flow of Lubricating Greases. Ind. Eng. Chem..

[48] Herschel W.H., Bulkley R. (1926). Konsistenzmessungen von Gummi-Benzollösungen. Kolloid-Zeitschrift.

[49] Hamze S., Cabaleiro D., Estellé P. (2021). Graphene-Based Nanofluids: A Comprehensive Review about Rheological Behavior and Dynamic Viscosity. J. Mol. Liq..

[50] Douglas J.F., Gasoriek J.M., Swaffield J., Jack L. (2005). Fluid Mechanics.

