# Supplementary material for: Rheology of Conductive High Reactivity Carbonaceous Material (HRCM)-Based Ink Suspensions: Dependence on Concentration and Temperature
Source: Nanomaterials (Basel). 2022 Dec 21;13(1):21. doi: 10.3390/nano13010021 (PMC9824156; doi:10.3390/nano13010021)
Supplement: Supplementary file 1 [file nanomaterials-13-00021-s001.zip › nanomaterials-2050696-supplementary.pdf]

nanomaterials

MDPI

Supplementary Materials

Flow curve comparison between 0.80 wt% HRCM ink and its components

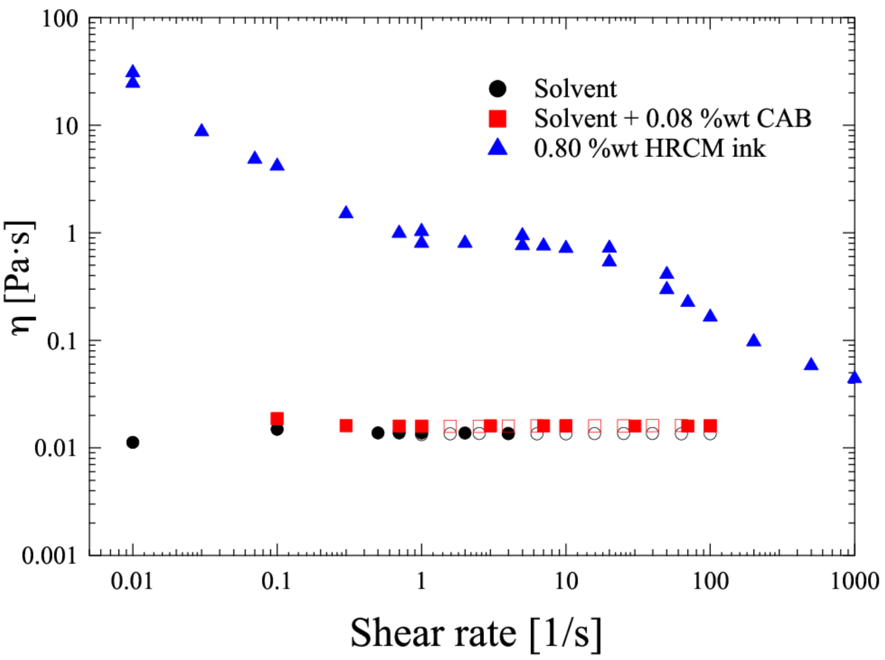

Figure S1. Flow curve comparison between 0.80 wt% HRCM ink and its components.

Standard deviation for some experimental replicates

Define

$$y_{ij} = \log_{10}(\eta_{ij}), \qquad i = 1,\dots,n, \qquad j = 1,\dots,m, \tag{SE.1}$$

the value observed at the i-th level of the experiment (temperature or shear rate) at the j-th replication.

At each level, the mean

$$\bar{y}_i = \frac{1}{m}\sum_{j=1}^{m} y_{ij}, \tag{SE.2}$$

and the normalized standard deviation

$$\sigma_i = \sum_{j=1}^{m}\sqrt{\frac{1}{m-1}\left(\frac{y_{ij}-\bar{y}_j}{\bar{y}_j}\right)^2}, \tag{SE.3}$$

were computed.

The value

$$\bar{\sigma} = \frac{1}{n}\sum_{i=1}^{n}\sigma_i \tag{SE.4}$$

was eventually computed.

nanomaterials

MDPI

Table S1. Computation of the standard deviation for some replicates of flow curve and temperature ramp measurements.

	Concentration	Temperature/Shear rate	Standard deviation
Flow curves	0.08 wt%	10 °C	0.24184
		20 °C	0.01591
	0.16 wt%	50 °C	0.01280
	0.40 wt%	5 °C	0.04436
		10 °C	0.04439
		20 °C	0.00989
	0.80 wt%	20 °C	0.01854
Temperature ramps	0.80 wt%	1 s^{-1}	0.03662
		10 s^{-1}	0.05460
